# The efficacy and cardiac toxicity of different‐dose pegylated liposomal doxorubicin in elderly patients with diffuse large B lymphoma

**DOI:** 10.1002/cam4.5280

**Published:** 2022-10-06

**Authors:** Li Li, Rongrong Chen, De Zhou, Jianai Sun, Lulu Wang, Lixia Zhu, Huafei Shen, Wanzhuo Xie, Xiujin Ye

**Affiliations:** ^1^ Department of Hematology The First Affiliated Hospital, College of Medicine, Zhejiang University Hangzhou Zhejiang China; ^2^ Program in Clinical Medicine School of Medicine of Zhejiang University Hangzhou Zhejiang China

**Keywords:** cardiotoxicity, comprehensive geriatric assessment, diffuse large B‐cell lymphoma, epirubicin, overall survival, pegylated liposomal doxorubicin, progression‐free survival

## Abstract

**Objectives:**

In order to explore the impact of pegylated liposomal doxorubicin (PLD) dose intensity on survival outcomes of newly diagnosed elderly patients with diffuse large B‐cell lymphoma (DLBCL), we performed a retrospective study to compare the efficacy and adverse effects of RCEOP (70 mg/m^2^), RCdOP (20–30 mg/m^2^) and RCDOP (30–45 mg/m^2^). The optimal PLD dose of patients with different clinical characteristics of subgroups was explored to provide a clue for the selection of clinical PLD dose.

**Methods:**

A total of 335 DLBCL patients (60–85 years old) who were newly diagnosed and completed at least four cycles of RCE(D)OP were selected. The patients were mainly divided into RCEOP (126 cases) (epirubicin 70 mg/m^2^), RCdOP (151 cases) (PLD 20–30 mg/m^2^) and RCdOP (58 cases) (PLD 30–45 mg/m^2^). The effects of different doses of PLD on clinical efficacy, cardiotoxicity and prognosis of patients were retrospectively analyzed. Subgroup analysis was performed to compare the clinical characteristics of different subgroups.

**Results:**

Our study showed that PLD and epirubicin had similar efficacy (overall survival (OS) *p* = 0.776; progression‐free survival (PFS) *p* = 0.959). RCDOP (30–45 mg/m^2^ PLD) group had a higher complete remission (CR) rate of 75.9% compared with RCdOP (20–30 mg/m^2^ PLD) group (P D vs. *d* = 0.018). In the overall population, there was no significant difference in survival between RCDOP and RCdOP groups (OS P D vs. *d* = 0.661; PFS P D vs. *d* = 0.212). In patients with underlying cardiovascular diseases, the PFS of the RCDOP group was significantly better than the RCdOP group (*p* = 0.043). Meanwhile, patients in the RCDOP group tended to have a better prognosis than those in the RCEOP group (OS: RCDOP vs. RCEOP *p* = 0.054, PFS: RCDOP vs. RCEOP *p* = 0.053). There was no significant difference in the incidence of cardiotoxicity and other adverse events among the three groups. For the low‐risk (age‐adjusted‐International Prognostic Index = 0/1) old patients without cardiovascular disease, RCdOP was considered a better strategy in OS (*p* = 0.020).

**Conclusion:**

In the general population, the CR rate in the RCDOP group was significantly higher than that in the RCdOP group (*p* = 0.018). For elderly DLBCL patients with cardiovascular disease, the effect benefit brought by the PLD dose was more obvious, and the PFS of the RCDOP group was significantly better than that of the RCdOP group (*p* = 0.043). Full dose of PLD is an efficient alternative in the treatment of patients with preexisting cardiovascular diseases.

## Graphical abstract

Generally, the complete remission rate of the RCDOP group was significantly higher than that of the RCdOP group. For elderly diffuse large B‐cell lymphoma patients with cardiovascular disease, the effect benefit brought by pegylated liposomal doxorubicin dose was more obvious, and the progression‐free survival of the RCDOP group was significantly longer than that of the RCdOP group (*p* = 0.043).

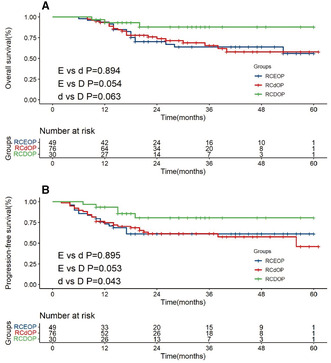

## 1 INTRODUCTION

Diffuse large B‐cell lymphoma (DLBCL) is the most common subtype of non‐Hodgkin's lymphoma (NHL), which accounts for approximately 30%–40% of all NHL.^1^ RCHOP regimen is currently the standard frontline treatment for DLBCL that can achieve complete remission (CR) in nearly 80% of patients.^2^ DLBCL frequently affects elderly people, about 40% of which happen in patients aged over 70 years.^3^ The older face unique challenges in the treatment of DLBCL.^4^ Bataillard et al. found that old‐fit patients can improve survival with higher relative dose intensity.^5^ However, drug toxicity and poor tolerance to chemotherapy limit the benefit of efficacy from the full‐dose RCHOP regimen. Moreover, the potential loss of efficacy is critical in elderly patients who are not feasible for chimeric antigen receptor T‐cell (CART) and autologous stem‐cell transplant.^6^

Anthracycline has been considered a cornerstone in the management of the disease while always accompanied by cardiac toxic side effects. The decision to reduce the anthracycline dose is a risk balance between toxicity and efficacy in clinical work, especially for older patients who may have additional cardiac risk factors. Pegylated liposomal doxorubicin (PLD), a kind of liposome formulation of doxorubicin, is small enough in size (80–90 nm) to selectively pass through the endothelium fenestrations of tumor blood vessels, minimizing the release in plasma and normal tissue.^7^ Therefore, PLD has a lower level of cardiotoxicity and “targeted” therapeutic effects for its unique pharmacology. It has been demonstrated that PLD has similar efficacy with an acceptable risk of cardiotoxicity in the therapy in DLCBL.^8^

The recommended PLD dose ranges from 20 to 45 mg/m^2^ based on previous studies.^8^, ^9^, ^10^, ^11^, ^12^, ^13^, ^14^, ^15^ However, it remains unclear whether the application of PLD can make more elderly patients benefit from full‐dose RCHOP chemotherapy and which group are more suitable for reduced doses of PLD. Therefore, we evaluate the efficacy and cardiac toxicity of different doses of PLD (20–30 and 30–45 mg/m^2^) versus epirubicin (70 mg/m^2^) in newly diagnosed elderly DLBCL patients and explore the optimal dose of PLD in different risk subgroups.

## 2 METHODS

### 2.1 Patients and data collection

**FIGURE 1:**
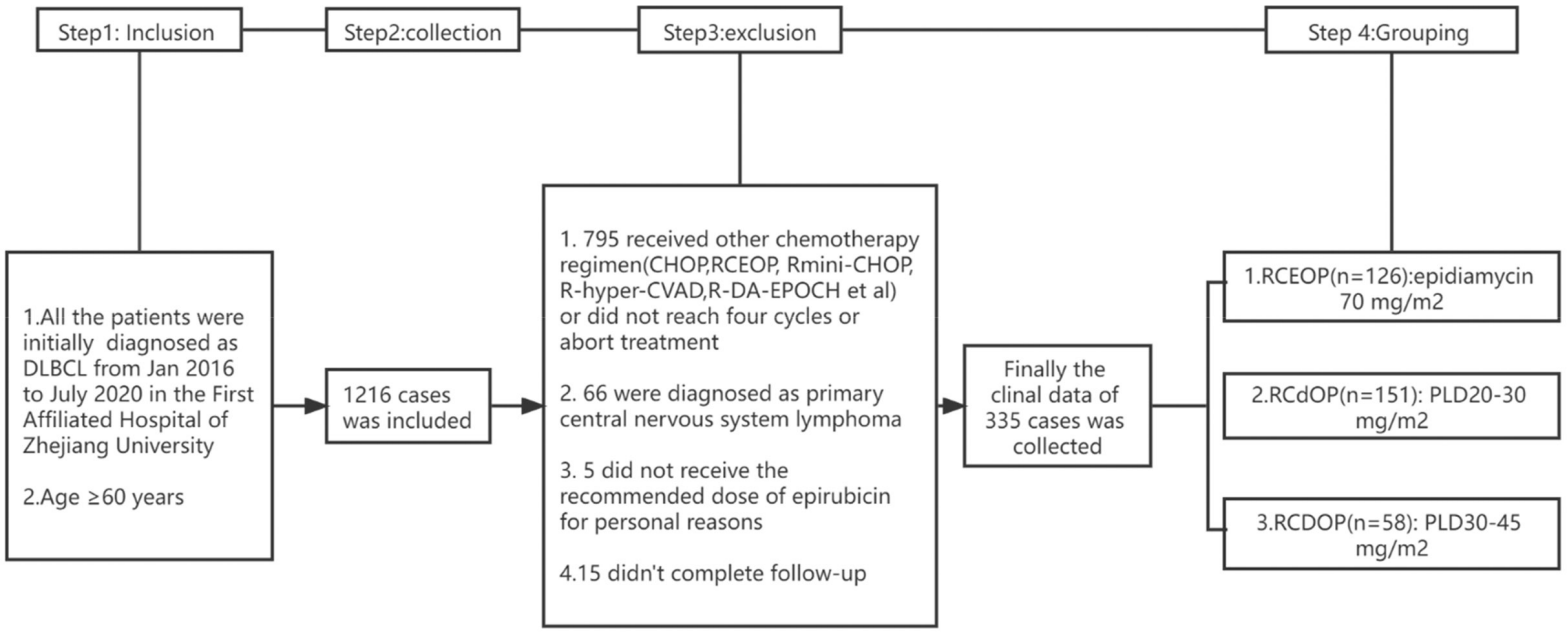
Flowchart of the patient enrollment process.

A total of 1216 patients who were newly diagnosed with DLBCL patients (aged 60–85 years) from different hematological disease centers in the First Affiliated Hospital, College of Medicine, Zhejiang University between January 2016 and July 2020 were retrospectively reviewed. Among these patients, 335 received at least four courses of the RCHOP regimen and completed follow‐up (Figure 1). All diagnoses were confirmed by histopathological staining (hematoxylin and eosin) and immunophenotyping according to the World Health Organization Classification. Clinical staging and diagnostic methods included clinical history, physical examination, computed tomography (CT) scan of the chest, abdomen and pelvis, full‐digital full‐body color Doppler ultrasonic diagnostic analyzer, positron emission computed tomography (PET‐CT), marrow aspirate and biopsy. In this study, patients were evaluated for clinical staging and efficacy by PET‐CT. Repeat echocardiography imaging was planned before every cycle and after treatment to assess the changes in the left ventricular ejection fraction (LVEF).

### 2.2 Treatment

The regimens consisted of rituximab 375 mg/m^2^ intravenously (i.v.) on day 1, cyclophosphamide 750 mg/m^2^ i.v., epirubicin 70 mg/m^2^ (RCEOP) i.v., or PLD 20–30 mg/m^2^ (RCdOP) [25.32 ± 2.51 (20.11–29.85)] i.v., or PLD 30–45 mg/m^2^ (RCDOP) [37.87 ± 2.83 (30.03–44.82)] i.v., and vincristine 1.4 mg/m^2^ (max. 2 mg/m^2^) i.v. on day 2 and prednisone 100 mg i.v./po on days 2–6 of each cycle. This regimen was repeated every 21 days. Median cycles were 6 (range, 4–8).

### 2.3 End points and assessments

In our study, the efficacy was mainly evaluated according to imaging response (CT) and metabolic response (PET/CT) according to modified Lugano 2014 criteria. PET‐CT will be used to assess response in 18‐fluoro‐deoxyglucose (FDG)‐avid histologies using Deauville 5‐PS standard. The degree of FDG uptake was compared with mediastinal blood pool and liver background. One score was no uptake, two scores were lower than mediastinum, three scores were between mediastinum and liver background, four scores were higher than liver background (less than three times), five scores were significantly higher than liver background (more than three times) or new lesions appeared.

Assessments of disease response were conducted 6–8 weeks after day 1 of the last cycle(“R‐CEOP”, “R‐CDOP” or “R‐CdOP”) at end of treatment response visit. The efficacy end points in our research included investigator‐assessed objective response rate (ORR = partial remission (PR) + CR), CR, progression‐free survival (PFS), overall survival (OS). Besides, PR, stable disease (SD) and progressive disease (PD) information was provided according to our original data. The compared results among the three study groups have been tabulated in Table 2.

The effect of drugs on cardiotoxicity was mainly evaluated by LVEF classified by the National Cancer Institute. Cardiac insufficiency grade 1 is asymptomatic and LVEF is < 20% lower than before chemotherapy. Grade 2 is asymptomatic and LVEF is ≥20% lower than before chemotherapy. Grade 3 is a mild cardiac failure with effective treatment. Grade 4 is severe or refractory heart failure.

Arrhythmia: Grade 0 is no arrhythmia event; grade 1 is asymptomatic and transient without treatment. Grade 2 is frequent or persistent, but does not require treatment; grade 3 arrhythmia events require treatment; grade 4 requires monitoring, or hypotension or ventricular tachycardia or ventricular fibrillation.

### 2.4 Statistical analysis

Continuous variables were expressed as median (range) and were compared using the independent sample the *t*‐test or Kruskal–Wallis test. Categorical variables are expressed in terms of quantity (percentage) and were compared using the chi‐square test or the Fisher exact test. Survival analysis using the Kaplan–Meier method and the differences between groups were compared using the Log‐rank test. PS matching (1v1) was performed using propensity scores (A. RCDOP vs. RCEOP; B. RCdOP vs. RCEOP), and the matching tolerance was 0.02. Differences between the comparative test results were considered significant if the two‐sided *p*‐value was <0.05. All statistical analyses were performed using SPSS 22.0 and R 4.1.2 The forest plot is made by Stata software.

## 3 RESULTS

### 3.1 Patient characteristics

From January 2016 to July 2020, 335 elderly patients (age range 60–85, male/female = 165/170) were analyzed in the study. Among them, 126 cases were treated with the RCEOP regimen (epirubicin 70 mg/m^2^), 151 cases were treated with the RCdOP regimen (PLD 20–30 mg/m^2^) and 58 cases with the RCDOP regimen (PLD 30–45 mg/m^2^). Age‐adjusted International Prognostic Index (IPI) (aa‐IPI) score 2–3 was in 48.4% of patients. About 75.8% of patients had a performance status score ≤2 and 67.2% of patients had Ann Arbor stage III or IV. There were no significant differences in the variables between the three groups (*p* > 0.05). The median follow‐up time was 25 months (range, 3–71 months). Eighteen (5.4%) patients were eventually treated with CART, and the follow‐up endpoint of these patients was set at CART events in order to avoid impact on survival analysis. Table 1 shows patient characteristics.

**TABLE 1:** The clinical characteristics of patients in RCEOP (*n* = 126), RCdOP (*n* = 151) and RCDOP (*n* = 58) groups

	RCEOP (*n* = 126)	RCdOP (*n* = 151)	RCDOP (*n* = 58)	*p* value
Age [years, median (range)]	67 (60–81)	69 (60–85)	67 (60–85)	0.147
Gender, *n* (%)				0.886
Male	60 (47.6)	75 (49.7)	30 (51.7)	
Female	66 (52.4)	76 (50.3)	28 (48.3)	
β2‐MG, *n* (%)				0.943
Normal	57 (45.2)	67 (44.4)	25 (43.1)	
Elevated	62 (49.2)	78 (51.7)	30 (51.7)	
NA	7 (5.6)	6 (4)	3 (5.2)	
Viral hepatitis B, *n* (%)				0.918
Yes	13 (10.3)	16 (10.6)	5 (8.6)	
No	113 (89.7)	135 (89.4)	53 (90.4)	
B symptoms, *n* (%)				0.400
Yes	41 (32.5)	61 (40.4)	21 (36.2)	
No	85 (67.5)	90 (59.6)	37 (63.8)	
Cell of origin, *n* (%)				0.417
GCB	36 (28.6)	47 (31.1)	24 (41.4)	
Non‐GCB	80 (63.5)	96 (63.6)	30 (51.7)	
NA	10 (7.9)	8 (5.3)	4 (6.9)	
Performance status (ECOG), *n* (%)				0.053
0–1	101 (80.2)	108 (71.5)	45 (77.6)	
2–4	25 (19.8)	43 (28.5)	13 (22.4)	
Ann Arbor stage, *n* (%)				0.776
I–II	39 (31)	50 (33.1)	21 (36.2)	
III–IV	87 (69)	101 (66.9)	37 (63.8)	
Age‐adjusted IPI, *n* (%)				0.212
0–1	70 (55.6)	70 (46.4)	33 (56.9)	
2–3	56 (44.4)	81 (53.6)	25 (43.1)	
Hypertension or cardiovascular disease				0.108
Yes	49 (38.9)	76 (50.3)	30 (51.7)	
No	77 (61.1)	75 (49.7)	28 (48.3)	

Abbreviations: ECOG, Eastern Cooperative Group; GCB, germinal center B‐cell; IPI, International prognostic index; NA, not available.

### 3.2 Response and survival in all patients and patients with cardiovascular disease

There was no statistical difference in the ORR rate, SD rate, PD rate and recurrence rate in RCEOP, RCdOP and RCDOP groups (Table 2). There are 93.7%, 88.1% and 93.1% cases achieving ORR in three groups. The RCdOP group has relatively high recurrence rate, but there is no statistical significance. The CR rate of RCDOP was statistically higher than the RCdOP group (*p* = 0.018).

**TABLE 2:** The response rate in RCEOP (*n* = 138), RCdOP (*n* = 69) and RCDOP (*n* = 61) group

	RCEOP (*n* = 126)	RCdOP (*n* = 151)	RCDOP (*n* = 58)	*p* value (E vs. d)	*p* value (E vs. D)	*p* value (d vs. D)
CR, *n* (%)	79 (62.7)	88 (58.3)	44 (75.9)	0.454	0.078	0.018*
PR, *n* (%)	39 (31.0)	45 (29.8)	10 (17.2)	0.836	0.051	0.065
ORR, *n* (%)	118 (93.7)	13388.1)	54 (93.1)	0.113	0.889	0.289
SD, *n* (%)	3 (2.4)	10 (6.6)	3 (5.2)	0.096	0.322	0.689
PD, *n* (%)	5 (4.0)	8 (5.3)	1 (1.7)	0.602	0.426	0.254
Relapse, *n* (%)	23 (9.5)	26 (19.5)	6 (11.1)	0.286	0.392	0.212

Abbreviations: PD, progressive disease; PR, partial remission; SD, stable disease; ORR, Objective Response Rate; CR, complete remission

* *p* < 0.05 was considered statistically significant.

**FIGURE 2:**
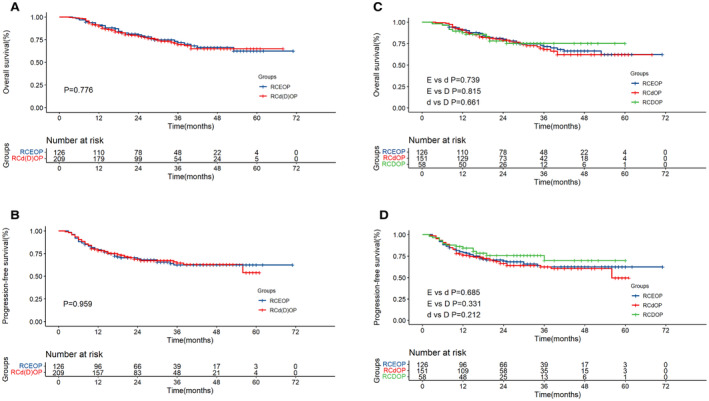
Overall survival (A) and progression‐free survival (B) survival curves of RCEOP, RCd(D)OP all patients. Overall survival (C) and progression‐free survival (D) survival curves of RCEOP, RCdOP and RCDOP groups in all patients.

Two‐years OS rate in RCEOP, RCdOP and RCDOP groups was 87.9%, 79.2% and 86.0%, respectively. And 2‐year PFS rate in RCEOP, RCdOP and RCDOP groups was 81.7%, 84.8% and 78.3%. There was no statistical difference in the OS and PFS in the RCEOP and RCd(D)OP groups (*p* = 0.776 and *p* = 0.959), which is consistent with the results of previous studies (Figure 2A, B). The RCDOP group showed a trend superior to the RCdOP group in PFS (*p* = 0.212, Figure 2D).

In order to avoid the influence of patient selection bias on efficacy, we conducted propensity score matching. The RCEOP group was taken as the reference group, and seven factors including B symptoms, cell of origin (COO) classification, Eastern Cooperative Oncology Group (ECOG) score, LDH, Ann staging, IPI score and cardiovascular disease were selected as covariables. The 1v1 matching method was adopted to screen corresponding patients from the RCdOP group and RCdOP group for matching. Finally, 108 patients in the RCdOP group found a match in the RCEOP group, and 56 patients in the RCDOP group found a match in the RCEOP group. The survival differences between matched RCdOP and matched RCEOP group, matched RCDOP and RCEOP group were further compared. After matching, there was almost no significant difference in the survival of the three groups. The results are presented in Figure 1.

In our study population, 155 patients had cardiovascular disease. The subgroup survival analysis was performed in this population in order to avoid bias caused by the choice of liposomal doxorubicin in clinical practice. Among them, 27 patients had heart diseases, including 19 patients with coronary heart disease, six patients with atrial fibrillation or other arrhythmias and two patients with other diseases. A total of 145 patients had hypertension and 16 patients had both hypertension and heart disease.

**FIGURE 3:**
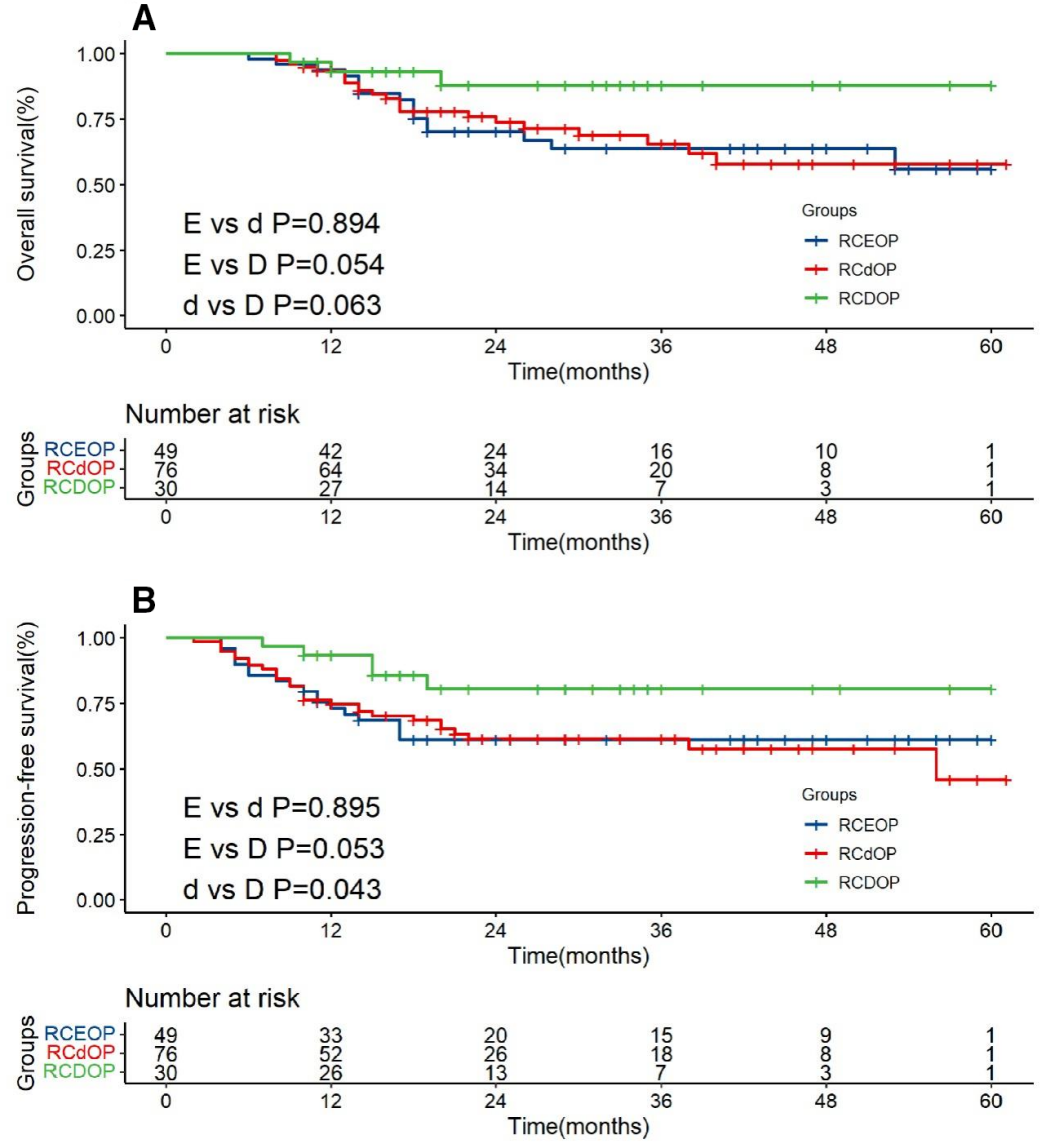
Overall survival (A) and progression‐free survival (B) survival curves of RCEOP, RCdOP and RCDOP groups in patients with cardiovascular diseases.

For patients with hypertension or heart disease, the RCDOP group has significant survival advantage compared with RCdOP in PFS (*p* = 0.043). Full dose of PLD is an efficient alternative in the treatment of patients with preexisting cardiovascular diseases. Meanwhile, patients in RCDOP group also have a better prognosis compared with RCEOP (*p* = 0.054 and *p* = 0.053, Figure 3A, B).

### 3.3 Subgroup analysis

To further explore the effects of different doses of pegylated liposomal doxorubicin on survival and the suitable groups of patients for treatment with reduced doses, the subgroup analysis was performed. According to whether age ≥ 70 years, ECOG ≥ 2, aa‐IPI scores ≥ 2, B symptoms, body mass index (BMI) ≥ 25,^16^ hypertension or cardiovascular disease, double‐express, elevated β2‐MG, elevated LDH, albumin ≤35 g/L, we divided the whole study population into subgroups.

**FIGURE 4:**
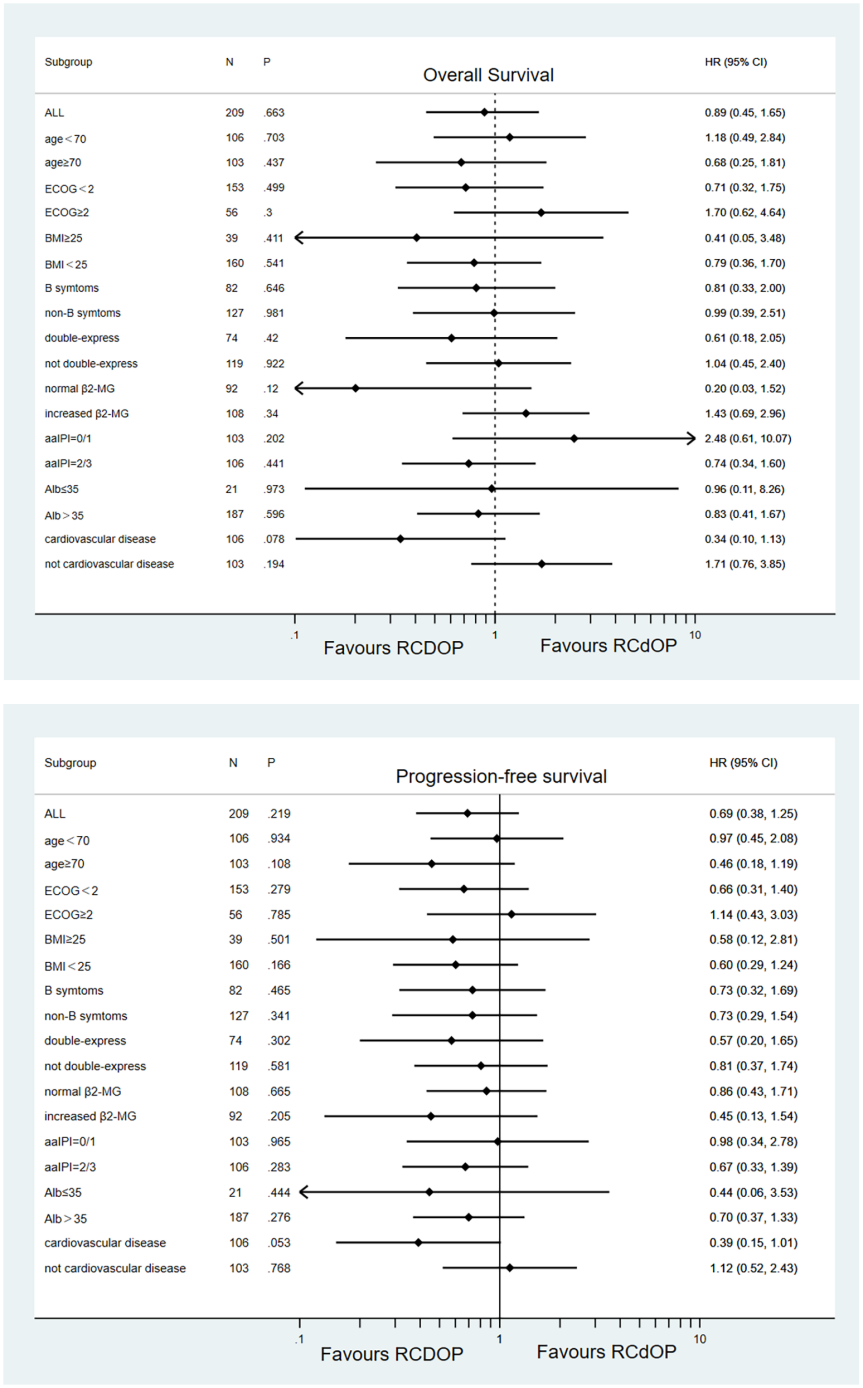
Subgroup survival analysis. Forest plot for potentially confounding factors for OS and PFS.

In different subgroups, survival of the treatment group with different doses of pegylated liposomal doxorubicin was compared, Figure 4. In OS, the RCDOP had survival advantage in normal β2‐MG subgroup and cardiovascular disease subgroup in OS (*p* = 0.12, *p* = 0.078). In PFS, the RCDOP had advantage in the ≥70 years subgroup, BMI <25 subgroup and cardiovascular disease group (*p* = 0.108, *p* = 0.166 and *p* = 0.053).

**FIGURE 5:**
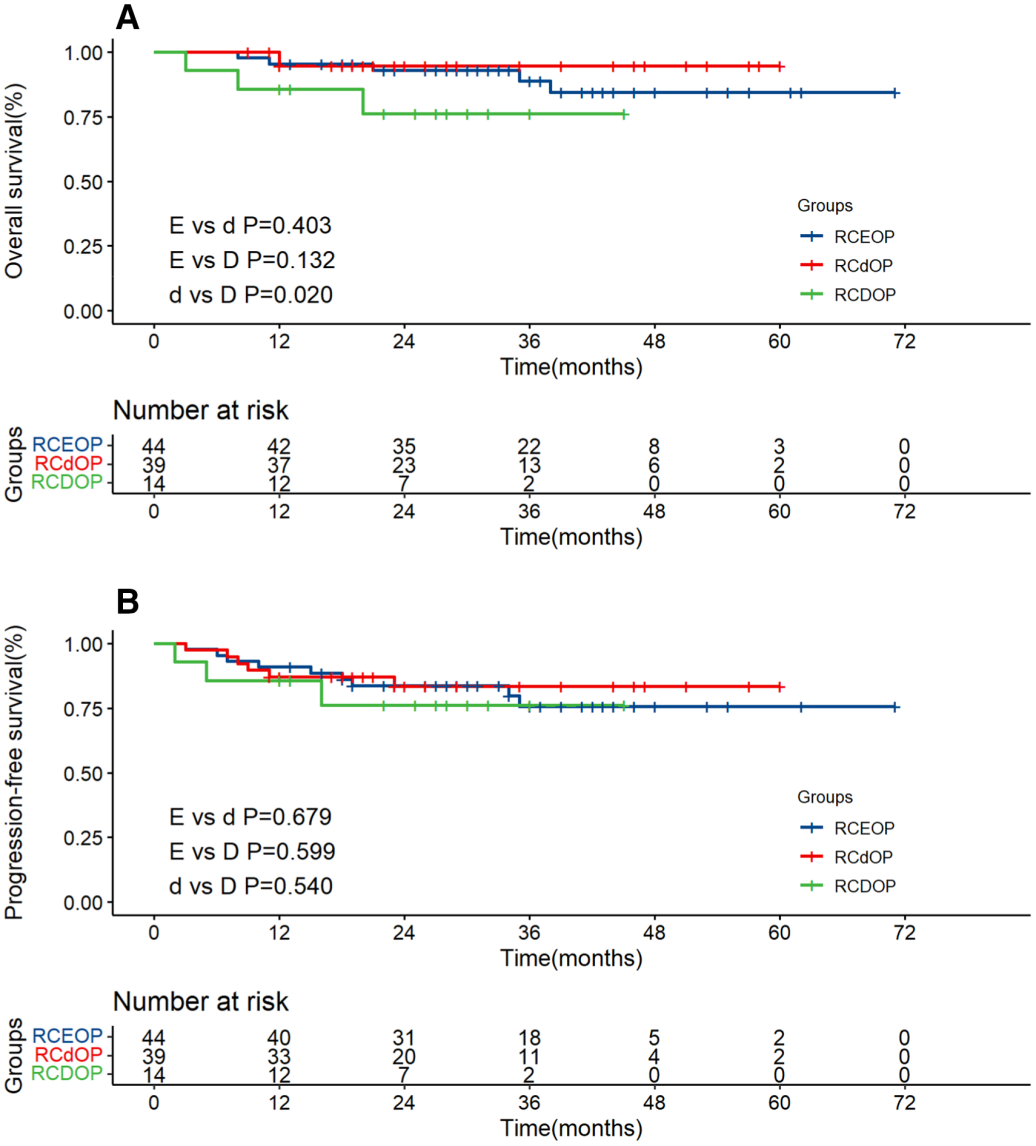
Overall survival (A) and progression‐free survival (B) survival curves of RCEOP, RCdOP and RCDOP groups in the low‐risk (aa‐IPI = 0/1) old patients without cardiovascular disease.

Because of its lower cardiotoxicity, more elderly adults could acquire better outcomes from full‐dose pegylated liposomal doxorubicin RCHOP regimens. However, in aa‐IPI = 0/1 and non‐cardiovascular disease groups, RCdOP seems showed a trend superior in OS (*p* = 0.202 HR (95%CI) 2.48 (0.61–10.07); *p* = 0.194 HR (95%CI) 1.71 (0.76–3.85)). Therefore, we further analyzed the differences in OS between RCdOP and RCDOP groups in aa‐IPI = 0/1 and non‐cardiovascular disease groups. In the patients with both aa‐IPI = 0/1 and non‐cardiovascular disease group, OS of the RCdOP group is statistically better than the RCDOP group (*p* = 0.02). This suggests that treatment regimens with reduced doses of PLD have an overall survival advantage in this population (Figure 5).

### 3.4 Analysis of cardiotoxic adverse events

**FIGURE 6:**
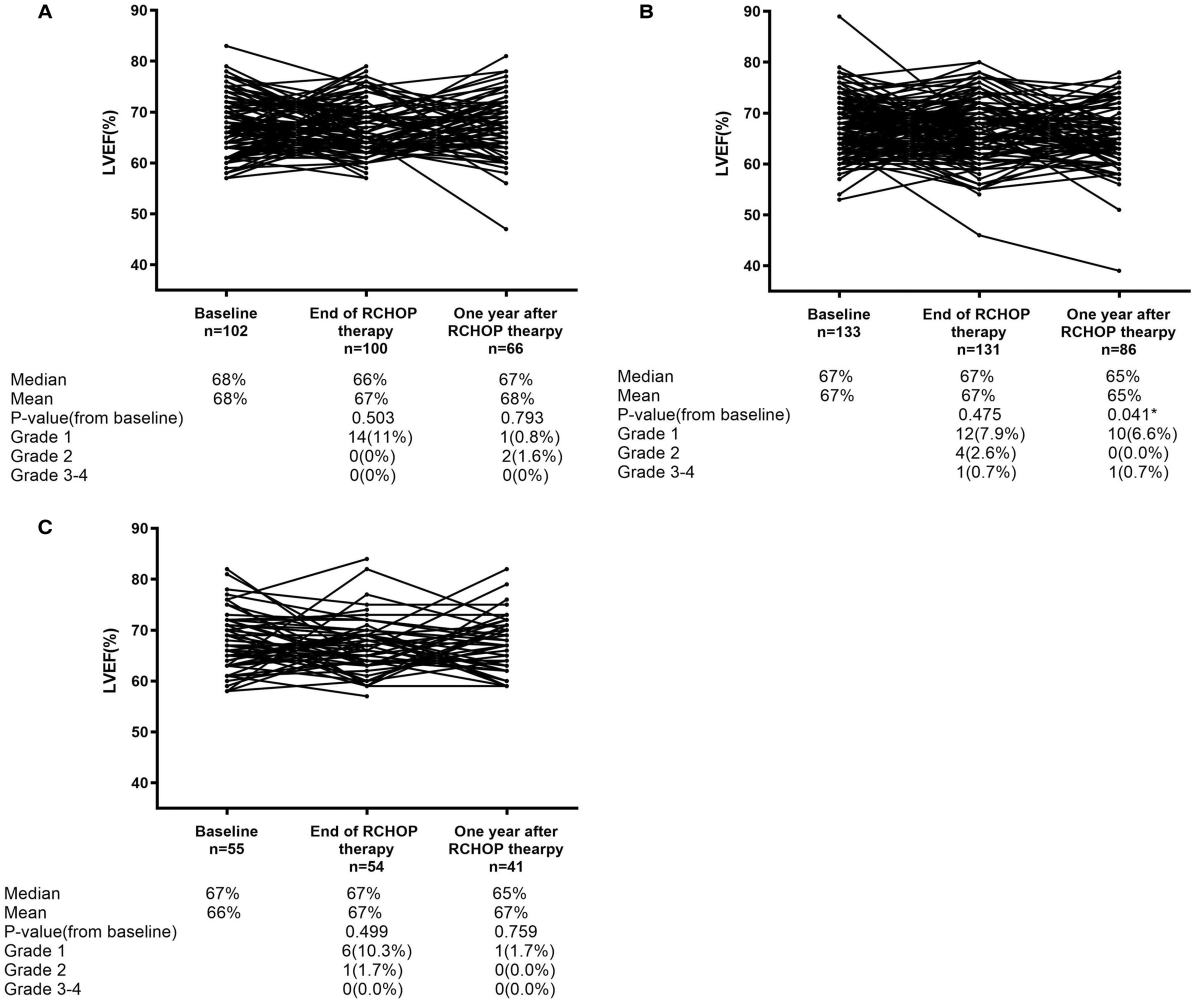
Change in left ventricular ejection (LVEF) during and after therapy (A) RCEOP, (B) RCdOP, (C):RCDOP.

The LVEF was assessed before therapy (*n* = 290), at therapy completion (*n* = 285) and 1 year after therapy (*n* = 193). Some patients lost part of LVEF data for they did not regularly review in our hospital. The LVEF findings are summarized in Figure 6. In a total of seven patients, LVEF was ≥20% lower than before chemotherapy (grade 2, RCEOP *n* = 2; RCdOP *n* = 4; RCDOP *n* = 1). And 71.4% (5/7) of these occurred at end of the treatment. One patient in the RCdOP group occurred grade 4 cardiotoxicity event during the treatment evaluations. The patient developed acute heart failure, acute renal failure, and rapid ventricular atrial fibrillation during treatment. The frequency of LVEF reduction and arrhythmia events in different subgroups is shown in Table 3. *p*1 value (RCEOP vs RCD[d]OP) and *p*2 value (RCdOP vs. RCDOP) have no significant meaning.

**TABLE 3:** Cardiotoxic adverse events

	Hypertension or cardiovascular disease (*n* = 155)			*p*1 value	*p*2 value
	RCEOP (*n* = 49)	RCdOP (*n* = 76)	RCDOP (*n* = 30)		
LVEF, *n* (%)				0.653	0.583
Grade 0	33 (67.3)	56 (73.7)	24 (80)		
Grade 1	5 (10.2)	7 (9.2)	4 (13.3)		
Grade 2	0 (0.0)	2 (2.6)	0 (0.0)		
Grades 3 and 4	0 (0.0)	0 (0.0)	0 (0.0)		
NA	11 (22.4)	11 (14.5)	2 (6.7)		
Arrhythmia, *n* (%)				0.389	0.136
Grade 0	22 (44.9)	26 (34.2)	18 (60.0)		
Grade 1	5 (10.2)	16 (21.1)	3 (10.0)		
Grade 2	2 (4.1)	6 (7.9)	2 (6.7)		
Grades 3 and 4	0 (0.0)	3 (3.9)	0 (0.0)		
NA	20 (40.8)	25 (32.9)	7 (23.3)		

	No hypertension or cardiovascular disease (*n* = 180)			*p*1 value	*p*2 value
	RCEOP (*n* = 77)	RCdOP (*n* = 75)	RCDOP (*n* = 28)		
LVEF, *n* (%)				0.219	0.933
Grade 0	53 (68.8)	58 (77.3)	23 (82.1)		
Grade 1	9 (11.7)	5 (6.7)	2 (7.1)		
Grade 2	0 (0.0)	2 (2.7)	1 (3.6)		
Grades 3 and 4	0 (0.0)	1 (1.3)	0 (0.0)		
NA	15 (19.5)	9 (12)	2 (7.1)		
Arrhythmia, *n* (%)				0.802	0.272
Grade 0	33 (42.9)	38 (50.7)	13 (46.4)		
Grade 1	11 (14.3)	8 (10.7)	6 (21.4)		
Grade 2	4 (5.2)	1 (1.3)	2 (7.1)		
Grades 3 and 4	1 (1.3)	1 (1.3)	0 (0.0)		
NA	28 (36.4)	27 (36.0)	0 (0.0)		

*Note*: *p*1value: RCEOP versus RCD(d)OP; *p*2value: RCdOP versus RCDOP.

Abbreviation: LVEF, left ventricular ejection.

## 4 DISCUSSION

The use of PLD in the RCHOP regimen has similar efficacy compared with epirubicin. Four previous studies have reported that PLD is an effective and well‐tolerated component for conventional doxorubicin in the “RCHOP” regimen (A phase II trial with 79 eligible elderly patients using PLD 40 mg/m^2.^ ^8^ In a study with 30 patients using PLD 30 mg/m^2^, 2‐year PFS and OS rate were 65.5% and 68.5%.^11^ A study with 25 patients aged over 70 with aggressive (stage III/IV) non‐Hodgkin's lymphoma using PLD 40 mg/m^2.^ ^17^ Our previous study with 103 patients aged between 60 and 75 years used 30–40 mg/m^2^ ^18^). Meanwhile, Schmitt et al. found that there was no difference between average event‐free survival in the subgroups treated with <15 mg/m^2^ and ≥ 15 mg/m^2^ in 21 NHL patients with cardiac risk factors.^12^ Non‐pegylated liposomal doxorubicin (NPLD) is another lipid form of doxorubicin. A meta‐analysis indicated that the replacement of doxorubicin with an equivalent dose of NPLD in the RCHOP regimen seems equally effective.^19^ However, the size of previous studies is relatively small and there has been few research focusing on the dose of PLD, especially in elderly patients with underlying heart disease. Therefore, we conducted a study of 335 elderly DLBCL patients treated with epirubicin or different doses of PLD.

There are mainly two key findings in our study. One finding is that the dose of PLD ranged 30–45 mg/m^2^ is recommended for most elderly DLBCL patients (age ≥ 60) generally. Especially, for patients with hypertension or heart disease, the RCDOP group has a significant survival advantage compared with RCdOP in PFS (*p* = 0.043). Our result revealed that intense PLD regimens confer survival benefits. In the survival comparison, RCDOP tends to be better than RCEOP and RCdOP in PFS (*p* = 0.331 and *p* = 0.212). Full dose of PLD is an efficient alternative in the treatment of patients with preexisting cardiovascular diseases. The enhanced dose–response effect of PLD may be explained by the reduced side effects of cardiac toxicity. In other words, in patients with cardiovascular disease, lower doses of traditional epirubicin can avoid toxic side effects, whereas lower doses of PLD are unnecessary and even less effective.

The other interesting finding is that RCdOP seems a better strategy for the low‐risk (aa‐IPI = 0/1) old patients without cardiovascular disease(*p* = 0.02). The dilemma of whether to treat elderly DLBCL patients with a full or reduced dose intensity of anthracyclines on RCHOP is often faced by clinicians. We did subgroup analyses to determine which groups of people are suitable for relatively low doses of PLD, which could provide clues to clinicians. The impact of RCHOP dose intensity on survival diminishes with increasing age.^5^ In low‐risk old patients without cardiovascular disease, our hypothesis is that “excess” doses of PLD do not confer further therapeutic survival benefits. In contrast, the possible side effects of PLD can have a negative impact on the patient's survival. Another study on the dose of RCHOP also suggested that patients with age >72 years and low aa‐IPI (0–1) had a better outcome when treated with R‐miniCEOP compared to those treated with RCHOP (HR = 0.13, *p* = 0.011).^20^ The very elderly patients (over the age of 80) may also be more suitable for reduced doses of PLD. However, we did not show their survival specifically for the limitation of the number of cases in our study. A prospective, multicentre, single‐arm study suggested that R‐miniCHOP should be the standard treatment for DLBCL patients older than 80 years.^21^

Comprehensive geriatric assessment (CGA) is a validated instrument evaluating functional age, generally including assessments of chronologic age, physical function, activities of daily living, instrumental (I) activities of daily living score (ADLs) and comorbidities. CGA also proved useful in judging patients for whom full‐dose RCHOP chemotherapy is unhelpful, and may even be detrimental. Tucci et al. found that 2‐year OS rate for patients deemed frail by CGA was only around 40%.^22^ Above all, age and risk stratification (aa‐IPI score) are primary considerations when deciding whether to reduce the PLD dose. CGA may be a good assessment tool to identify a “fit” patient. Further research needs to be performed to develop a complete scoring model.

In this study, the use of PLD did not show an advantage in reducing cardiac toxicity. The possible reasons are as follows: First, cardiotoxicity can be classified as acute, subacute, and chronic events. The median follow‐up time of our research was 25 months, when evaluating adverse events recorded only acute (occurred in the treatment of a few hours or within 1 week) and subacute (1 year after treatment of cardiac adverse events). Chronic cardiotoxic events require long‐term follow‐up and observation. Moreover, the development of anthracycline cardiotoxicity has a cumulative drug effect. Studies have shown that the incidence of cardiotoxic events increased significantly with the lifetime dose of adriamycin above 450–550 mg/m^2^.^23^ Another study showed that the incidence of congestive heart failure was 1.7% at a cumulative dose of 300 mg/m^2^, 4.7% at 400 mg/m^2^ and 15.7% at 500 mg/m^2^.^24^

In our study, the incidence of acute cardiotoxicity is significantly higher than that of subacute cardiotoxicity, and most acute cardiotoxicity events occur during or after treatment. Therefore, clinical management during the treatment is very important. Patients using PLD in our hospital may have more heart disease risk factors, but the difference was not statistically significant. In order to avoid bias, we conducted subgroup analysis on cardiotoxic events. There was no significant difference in the probability of cardiotoxic events among the three groups. There was one event (1/209) of grades 3 and 4 adverse cardiac and four events of (4/209) grades 3 and 4 arrhythmias were recorded in patients treated with PLD. In a previous study, during the 78 cycles (21 patients) applied, only one patient occurred acute cardiotoxicity.^12^ Overall, liposome‐encapsulated formulations have a reduced cardiotoxicity and preserved antitumor efficacy, which was confirmed by recent data from clinical trials.^25^, ^26^

To discuss the treatment of the elderly as a whole. Most elderly patients are not suitable for autologous stem‐cell transplant and have limited access to CART therapy. Therefore, the efficacy of initial chemotherapy is critical in these elderly patients.^6^ Though the adjusted RCHOP therapy regimen (R‐miniCHOP, R‐miniCEOP, dose‐adjusted etoposide, prednisone, vincristine, cyclophosphamide, doxorubicin, rituximab [DAEPOCH‐R] and rituximab, gemcitabine, cyclophosphamide, vincristine, prednisone [RGCVP]) and a combination of novel drugs (lenalidomide, obinutuzumab and ibrutinib) have emerged, RCHOP is still the standard first‐line treatment with relatively good efficacy. It is still necessary to explore the dosage and efficacy of liposomal doxorubicin and establish a more complete risk stratification. Elderly people should be more focused on the prevention and treatment of complications, pay more attention to the quality of survival. Toxicity and efficacy should maintain a balance in the treatment of elderly patients with DLBCL.

Our study still had some limitations. Since this was a retrospective study, although there was no statistically significant difference, there could be selection bias in the clinic. Moreover, we were not able to conduct CGA for all patients, so this important research factor was not included in the analysis. In addition, due to the relatively short follow‐up time, we did not conduct a long‐term comprehensive cardiac assessment and other toxic side effects (infections, hand–foot syndrome and neutropenia).

## 5 CONCLUSION

Overall, PLD is as effective as epirubicin with acceptable cardiotoxicity, and its use makes it possible that more elderly patients could benefit from full‐dose anthracycline therapy. For patients with hypertension or heart disease, the RCDOP group has significant survival advantage compared with RCdOP in PFS (*p* = 0.043). However, RCdOP seems a better strategy for the low‐risk (aa‐IPI = 0/1) old patients without cardiovascular disease (*p* = 0.02).

## AUTHOR CONTRIBUTIONS

Li Li and De Zhou conceived and supervised the study; De Zhou and Rongrong Chen designed experiments; De Zhou, Lixia Zhu, Lulu Wang, Jianai Sun, Xiudi Yang, Jingjing Zhu, Xiaolong Zheng performed experiments; De Zhou and Rongrong Chen analyzed data; De Zhou, Rongrong Chen and Lixia Zhu wrote the manuscript. All authors reviewed the results and approved the final version of the manuscript.

## Funding information

This study was supported by the Youth Program in Natural Science Project of Zhejiang Province (LQ16H160009). The funders had no role in study design, data collection and analysis, decision to publish or preparation of the manuscript.

## CONFLICT OF INTEREST

The authors declare that they have no competing interests.

## ETHICS APPROVAL AND CONSENT TO PARTICIPATE

The Ethics Committee of the First Affiliated Hospital, College of Medicine, Zhejiang University, approved this study.

## Supporting information


Figure S1

## Data Availability

Some or all data, models used during the study are available.
