# Supplementary material for: The efficacy and cardiac toxicity of different‐dose pegylated liposomal doxorubicin in elderly patients with diffuse large B lymphoma
Source: Cancer Med. 2022 Oct 6;12(4):4184–94. doi: 10.1002/cam4.5280 (PMC9972167; doi:10.1002/cam4.5280)
Supplement: Supplementary file 1 — Figure S1 [file CAM4-12-4184-s001.docx]

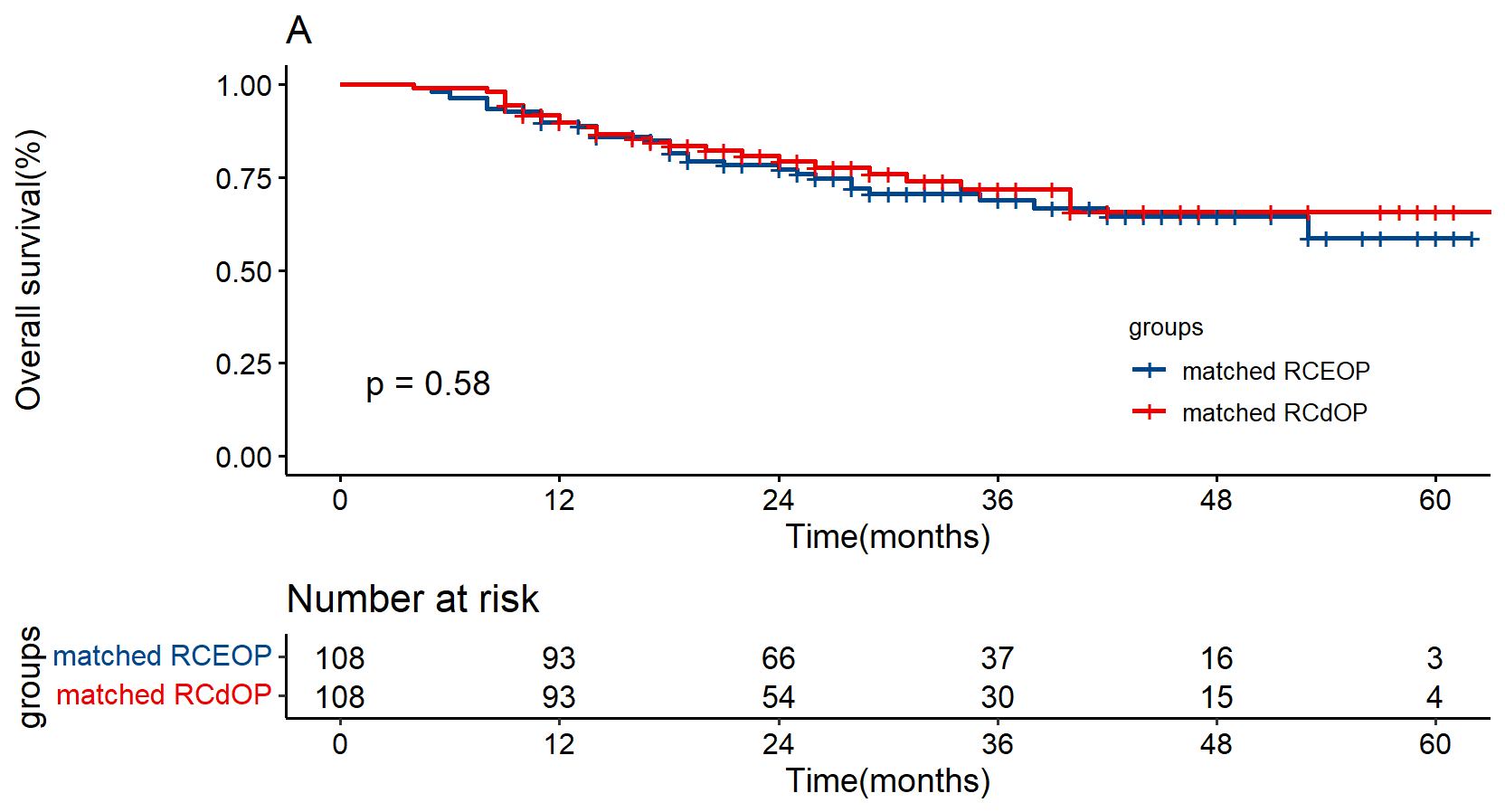

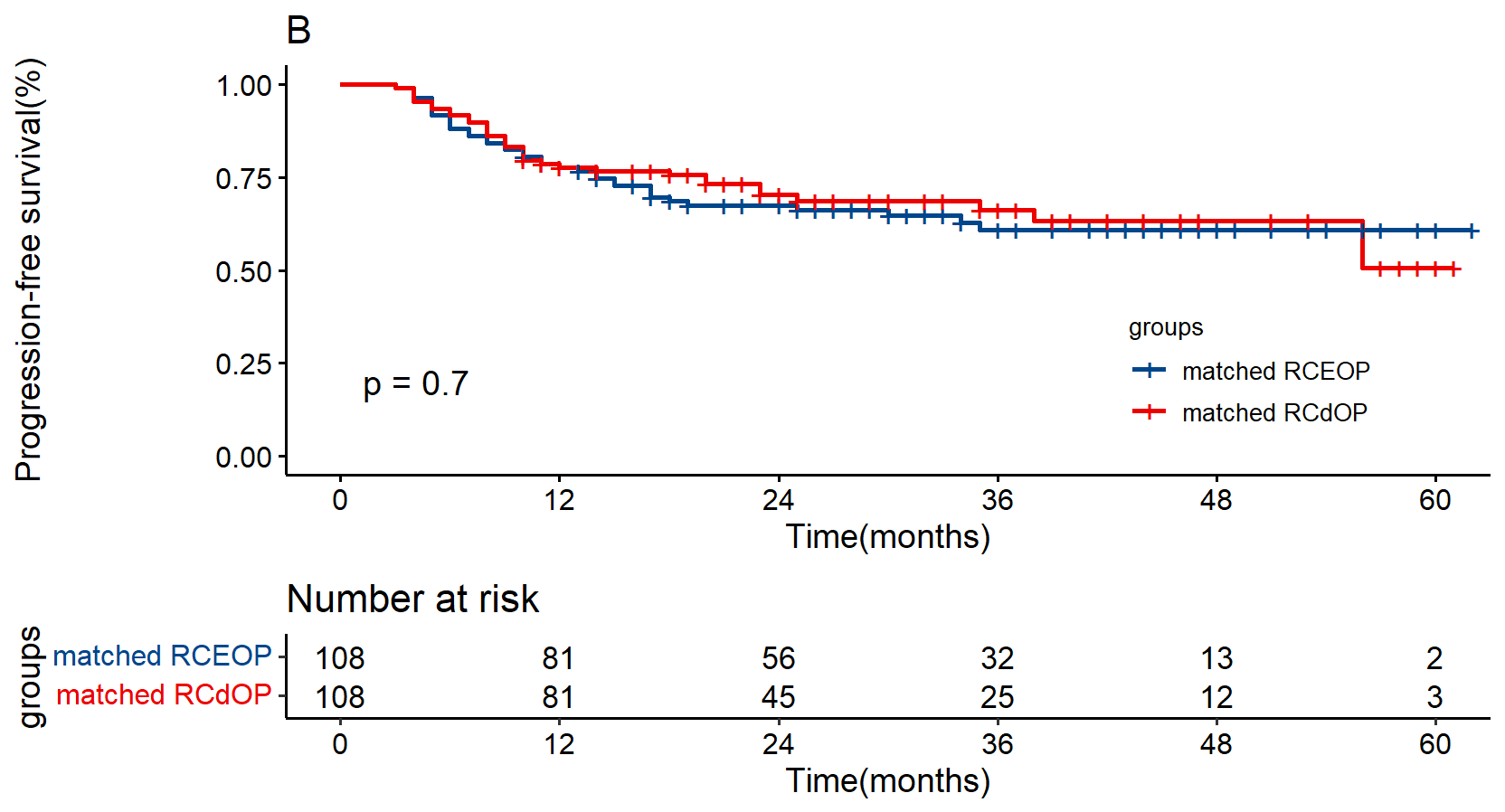

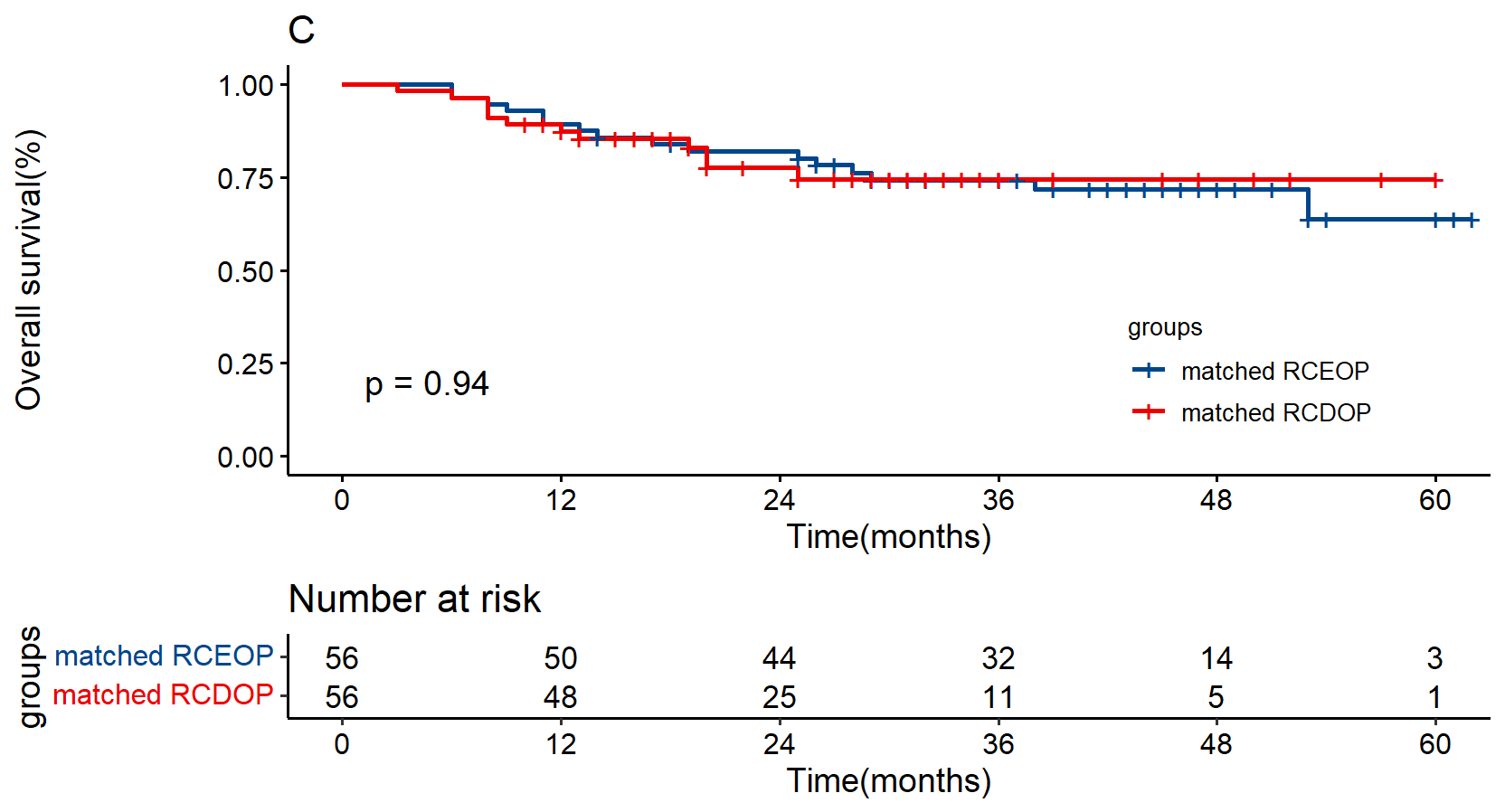

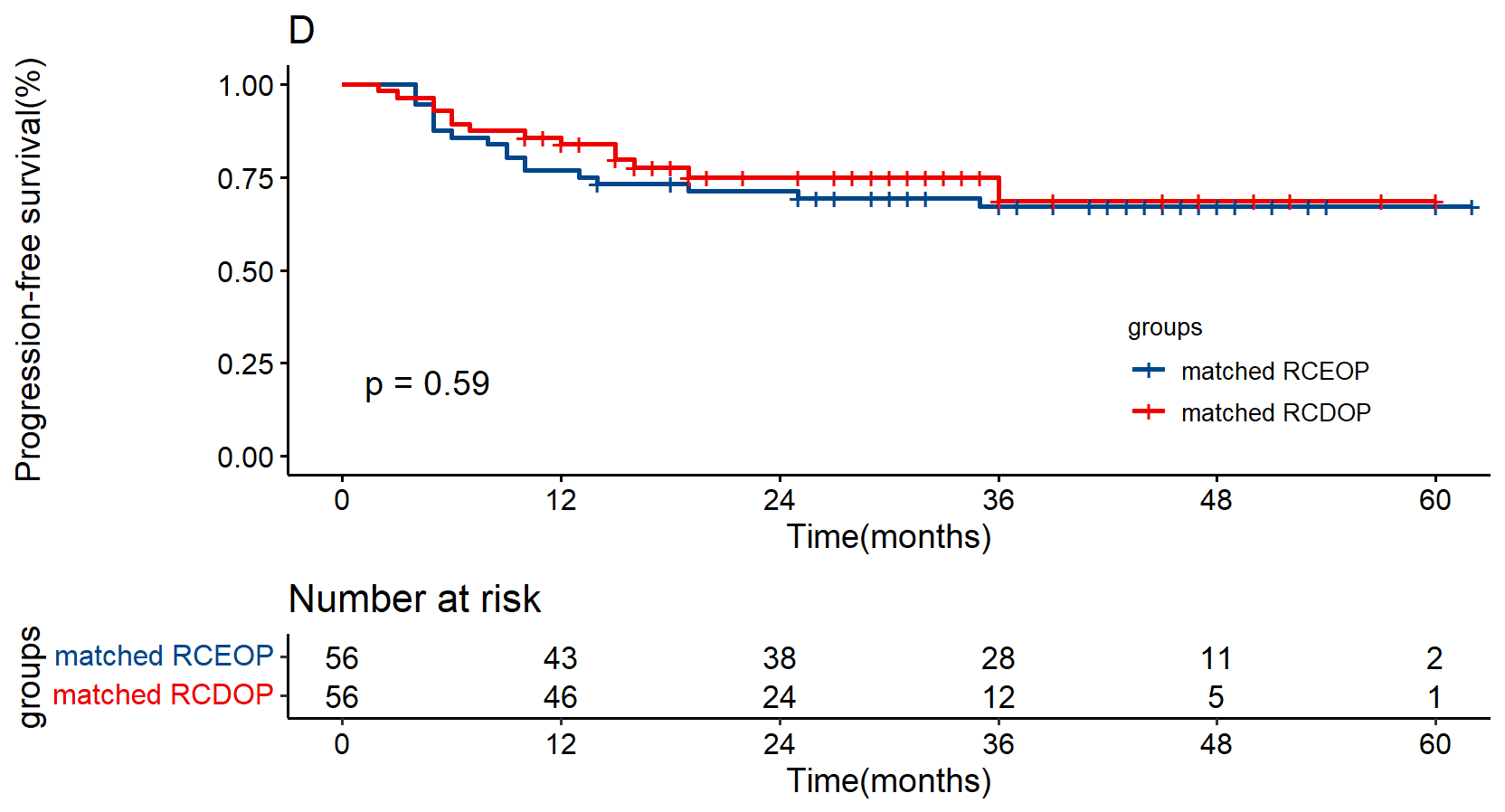


Supplementary figure :Overall survival(A) and Progression-free survival (B) survival curves of matched RCEOP and RCdOP group；Overall survival(C) and Progression-free survival (D) survival curves of matched RCEOP and RCDOP group
